# Editorial Boasting

**Published:** 1888-09

**Authors:** 


### "EDITORIAL BOASTING."

Under the caption of "Editorial Boasting," the editor of *Items of Interest* unloads himself of considerable pent-up feeling. He criticises a straightforward business announcement of the International Dental Journal Association, evidently failing to recognize in it a living protest against the very class of journalism his represents. The production was in no sense a personal editorial, but voiced the sentiment of a syndicate composed of some of the very best men in the profession, and too strong to be lightly sneered at by the organ of a house that depends upon the profession for its support. We occupy an entirely different position from the trade journals; with us, independent journalism is first, and trade a secondary consideration, while with them the opposite is the case, or else we have entirely mistaken the business sagacity of the men who stand behind them. It would seem, however, that in this instance ordinary commercial foresight should have suggested a different policy upon the part of the *Items*.

It is no fault of ours that we find ourselves the editorial mouthpiece of the strongest syndicate ever organized in the dental profession, neither does the fact of its being International in character in any way detract from its strength. The present organization is simply the outgrowth of the advances made by the dental profession during the present decade, and to which the better class of trade journals have contributed their quota. No organ, in the time it has been before the profession, has done more than THE INDEPENDENT PRACTITIONER. Its columns have throughout reflected a high class of literature, and its original communications have been more widely quoted than those of any other journal; although, I am sorry to say, not always been given due credit. An instance of this latter is found in the very number of the *Items* in which we are criticised. In this case a full page was taken bodily from the June number of THE PRACTITIONER without a line of credit being given in the text as to where it had previously appeared. In a journal made up almost exclusively of clippings, as is the *Items,* it must sometimes be rather confusing to give credit to all the matter without getting them mixed; and we could overlook small slips were it not for the lordly assumption of age and experience assumed by its editor, and the manner in which we are told to "bide our time and learn what it is to edit a dental journal." We confess that our experience in "tailoring" has been rather limited. We have always stood upon our own base, and do not intend to depart from first principles, now that we have *dared* assume the editorial chair. We do not intend that our journal shall cling like a barnacle to the skirts of other journals that have the energy to secure original matter for their columns, but shall always try to have something new and of interest to the profession. If two or three of the leading journals should decide to copyright their journals, our would-be critic would find his laborious task of using the shears taken away, and be compelled to look elsewhere for his material.

In the article in question we made no mention of names, consequently it is rather amusing to see how readily the *Items of Interest* has put on the shoe of "trade journalism," and not only accepted the very modest definition we gave of their purpose and mission, but has added an additional clause, viz., "Mere advertising circulars for local houses." We are willing to let every one describe their own wares, knowing full well that if they will do so they can do it much better than any one else. On the whole, we really cannot see what there was in the simple announcement of an independent syndicate to call forth such an editorial, unless the shoe did really pinch. If that is the case, we would humbly, in view of our "inexperience" in dental, although not in other journalism, suggest to our honored and senior brother of the quill to enlarge the foundation upon which he stands, and take a position among the leading journals instead of depending upon the efforts and enterprise of others for his matter.

